# Prevalence of Traditional Risk Factors in First-Degree Relatives of Patients With Established Cardiovascular Disease

**DOI:** 10.7759/cureus.39061

**Published:** 2023-05-15

**Authors:** Poojan J Prajapati, Vatsa Bhavsar, Dakshey Bhatt, Ashwati Konat, Saujas Shah, Vatsal Zapadia, Dhruvam Nanavati, Shailee Shroff, Neel Vora, Kamal Sharma

**Affiliations:** 1 Internal Medicine, B. J. Medical College, Ahmedabad, IND; 2 Internal Medicine, GMERS (Gujarat Medical Education and Research Society), Himmatnagar, IND; 3 Department of Zoology, Biomedical Technology and Human Genetics, Gujarat University, Ahmedabad, IND; 4 Internal Medicine, Gujarat Cancer Society (GCS) Medical College, Ahmedabad, IND; 5 Cardiology, Dr. Kamal Sharma Cardiology Clinic, Ahmedabad, IND

**Keywords:** risk scores, traditional risk factors, attendees of patients, who/ish risk prediction chart, cardiovascular disease

## Abstract

Introduction: World Health Organization (WHO)/International Society of Hypertension (ISH) risk prediction charts are useful for predicting 10-year combined myocardial infarction and stroke risk (fatal and non-fatal). Hence the current study was conducted to assess the 10-year risk of cardiovascular disease among adults in Ahmedabad, India.

Aims: The primary aim of the study was to assess the cardiovascular risk among first-degree relatives of patients attending the outpatient clinic. Also, to create awareness regarding assessment of cardiovascular risk among the studied group.

Methods and materials: A cross-sectional study was carried out among 372 first-degree relatives of patients at an out-patient cardiology clinic present in Vadaj, Ahmedabad. The WHO/ISH risk prediction chart for South-East Asia Region D (SEAR D) was used for calculating the 10-year cardiovascular risk.

Results: A maximum (80.10%) of the study participants were in the low-risk (<10%) category followed by 8.33% for moderate-risk (10-20%), 7.25% for moderately high-risk (20-30%), 2.42% for high-risk (30-40%) and 1.88% for very high-risk (>40%).

Conclusion: WHO/ISH risk prediction charts provide a quick and effective way to assess and categorize the population in a low-resource setting which in turn helps in delivering focused intervention to the high-risk groups.

## Introduction

Cardiovascular diseases (CVD) are a major public health issue worldwide, with a higher incidence and severity in developing countries. It is estimated that CVD contributed to an estimated death of 17.9 million globally in the year 2019 [1]. Out of all CVD-related fatalities, 85% were related to heart disease and stroke. Out of all non-communicable disease-related death, CVD is the highest contributor followed by cancer, chronic respiratory diseases and diabetes [2]. As per estimation by Zhao et al., 27.4% of all fatalities in India are attributable to CVD, with 50.6% of these deaths occurring in less than 70 years of age [3]. Norman et al. predicted that India's CVD burden would quadruple in the next two decades due to the ongoing demographic and epidemiological change experienced by middle- and low-income nations [4]. CVD is usually the result of a complex interface of various factors related to hereditary, socioeconomic, personal, medical, environmental, and healthcare. This morbidity and mortality can be reduced substantially by undertaking population-based initiatives and providing accessible, affordable and cost-effective medications for patients with pre-existing diseases as well as those who are prone to them [5].

The purpose of the current study was to evaluate the prevalence of cardiovascular risk and improve CVD awareness among first-degree relatives of patients with age 40 years and above, using World Health Organization (WHO)/International Society of Hypertension (ISH) risk prediction charts. For this purpose, data on the identification of risk factors and the relative risk for myocardial infarction (MI) and stroke from each of the 14 designated WHO epidemiologic sub-regions were used to construct the two sets of WHO/ISH CVD risk prediction charts (with and without cholesterol). The 10-year risk of either a fatal or non-fatal major cardiovascular event can be estimated using WHO/ISH risk prediction tables based on the patient’s age, sex, blood pressure, smoking status, total blood cholesterol, and diabetes mellitus status (myocardial infarction or stroke) [6-8]. Estimation of cholesterol levels in the blood is a quick and reliable option for assessing preliminary CVD risk. However, in many developing and middle-income countries, laboratories are not easily accessible to patients and their first-degree relatives. In the absence of blood cholesterol testing, the WHO/ISH risk prediction table for South-East Asian Region D still can be utilized [7]. The WHO/ISH charts may help clinicians in safeguarding the patient’s life along with improvement in the patient’s quality of life as well as the extension of life. Due to their ease of use and affordability, these charts may serve as a screening tool for predicting cardiovascular events [9].

## Materials and methods

Study design and settings

The site for the cross-sectional investigation was a cardiology clinic at Vadaj, Ahmedabad, Gujarat, India. The study population included first-degree relatives of patients treated at the outpatient department (OPD) of the cardiology clinic. The study was approved by Sanjeevani Superspeciality Hospital with approval number CVA-01/23. The inclusion criteria included first-degree relatives of patients with age ≥40 years, willing to participate and being aware of their diabetic status. First-degree relatives of patients who were <40 years of age, unwilling to participate, and not aware of their diabetic status and past history of MI were excluded. To calculate the minimum sample size, the following formula [10] was used:

$N=(Z^2*P*Q)/d^2$

Where,

N = Number of participants

Z = 1.96 for a 95% confidence interval

P = Prevalence of significant cardiovascular risk (10.6%) [11]

Q = 1-P

d = 0.05.

The minimum sample size obtained was 139. The original sample size of 100 was raised to 167 considering a probability of 20% of non-response out of the total response. A total of 372 people met the inclusion and exclusion criteria using the sample selection method.

Data collection

After obtaining verbal consent, data were collected using a pre-designed questionnaire from patients’ first-degree relatives aged ≥40 years. The pre-testing of the questionnaire was carried out using 10 participants followed by a study for all 372 individuals including blood pressure measurements. Socio-demographic variables collected included age, sex, education, religion, marital status, members of household, employment and income. Other details collected include family history, past history, diabetic status, diet, physical, smoking status, alcohol consumption and tobacco consumption. A digital sphygmomanometer was used to measure their blood pressure. The participant sat straight with one arm on a table and the measurement was taken at the upper right arm. Three individual readings were taken, each at a 60-second interval. The mean reading was determined by calculating the average values of the previous three readings. India is included in the South-East Asia Region D of the WHO/ISH risk prediction chart, which is used to forecast fatal/non-fatal cardiovascular events 10 years out (in contexts where blood cholesterol cannot be measured). Gender, diabetes, co-morbidity, smoking status, age, and blood pressure levels were among the characteristics used to predict CVD risk during a 10-year period. The chart divides people into five categories based on their level of risk: low (<10%), moderate (10% to <20%), moderate-high (20% to <30%), high (30% to <40%), and extremely high (>40%). Risk for CVD is shown on a scale as green (risk 10%), yellow (10% to 20%), orange (20% to 30%), red (30% to 40%), deep red (risk 40%) [6,8]. Risk scores were calculated after stratifying the collected data. Table 1 shows the following operational definitions.

**Table 1:** Operational Definitions ISH: International Society of Hypertension, ESC: European Society of Cardiology, ESH: European Society of Hypertension

Terms	Operational Definitions
Education status	None (no schooling), primary (up to 6th standard), secondary (up to 12th standard) and tertiary (college education)
Family History	Hypertension, Diabetes, Myocardial Infarction, Hypercholesterolemia, Stroke and Angina in blood relatives
Diabetic status	This was informed by the participant. Diabetics also included those using oral hypoglycemic medications or insulin for control of blood sugar levels
Smoking status	Smoker (if they reported smoking at the time of the survey), Former smoker (if they reported quitting smoking less than a year before)
Alcohol consumption	Alcoholics (self-reported as consuming more than 4 drinks in a day for men and more than 3 drinks in a day for females), Non-alcoholics (self-reported as not drinking alcohol or stopped drinking within the last year)
Tobacco consumption	Tobacco user (someone who uses tobacco regularly) or Tobacco non-user (not used tobacco at all or someone who has quit within the last year)
Physical activity status	Daily (at least 30 minutes daily), occasional (less than 5 times a week) and none. Moderately intense aerobic exercises such as walking, jogging, running, dancing, cycling, swimming etc were included.
Systolic blood pressure	The data was stratified with the interval of 20 mmHg into 5 groups. Based on ISH and ESC/ESH guidelines participants with systolic blood pressure >140 mmHg were considered hypertensives [12-14]
Diastolic blood pressure	The data was stratified with an interval of 10 mmHg. Participants with diastolic blood pressure >90 mmHg were considered hypertensives [12-14]

The participants were divided into four risk categories based on the color scheme charts: low (<10%), moderate (10% to <20%), moderately high (20% to <30%), high (30% to <40%), and very high (>40%) [14].

WHO/ISH Risk Score

Individuals were assigned to one of four risk groups based on the percentage of the population at each level, as shown by the color scheme: low (<10%), moderate (10% to <20%), moderately high (20% to <30%), high (30% to <40%), or extremely high (>40%) [6].

Data analysis

Excel 2016 software (Microsoft, Redmond, WA, USA) was used for data entry followed by data analysis using Statistical Package for Social Sciences (SPSS) version 23 (IBM Corp., Armonk, NY, USA). Variables used to summarize continuous data were mean, standard deviation, and range whereas frequencies and proportions were used to summarize categorical variables. People's distributions within CVD risk groups were analyzed using a chi-square test to find out if there was any correlation between demographic, clinical, and medical characteristics. The level of significance was set at p<0.05.

## Results

Of the total 372 participants, 73.92% (275) were males and 52.15% of the participants were between the ages of 40 to 49 years. Participants' marital status was reported in 99.63% of cases. The average age of the participant in the study was 51.2±9.32 years. Both the average systolic and diastolic blood pressures were high, with values of 133.59±16.87 and 84.85±10.76 mmHg, respectively. It was revealed that systolic blood pressure >140 mmHg was more common in males (32.72%) than in females (20.61%), with an overall prevalence of 29.56% among the participants. While the overall prevalence of diastolic blood pressure >90 mmHg was found to be 25.80% with a higher prevalence among males (29.09%) than in females (16.49%). Overall, the prevalence of diabetes mellitus was 11.82%, with males being 2.7% more likely to have diabetes mellitus compared to females (12.72% for males; 9.27% for females). Smokers and alcoholics constituted 5.37% and 2.6% of the studied population respectively. Smokers (7.27% male versus 0% female) and alcoholics (3.63% male versus 0% female) were found to be more prevalent in males (Table 2).

**Table 2:** Demographic details of the participants *(p-value<0.05 shows significant difference) WHO: World Health Organization, ISH: International Society of Hypertension, BP: blood pressure

Characteristics	Males N=275	Females N=97	Total N=372	P value
Age (in years)				
40-49	144 (52.36%)	50 (51.54%)	194 (52.15%)	0.24
50-59	65 (23.63%)	32 (32.98%)	97 (26.07%)	
60-69	50 (18.18%)	14 (14.43%)	64 (17.20%)	
70-79	16 (5.81%)	1 (1.03%)	17 (4.56%)	
Marital status				
Married	274 (99.63%)	96 (98.96%)	370 (99.46%)	0.093
Unmarried	1 (0.36%)	1 (1.03%)	2 (0.53%)	
Religion				
Hindu	267 (97.09%)	95 (97.93%)	362 (97.31%)	0.58
Muslim	7 (2.54%)	2 (2.06%)	9 (2.42%)	
Christian	1 (0.36%)	0	1 (0.26%)	
Education				
No formal schooling	2 (0.72%)	2 (2.06%)	4 (1.07%)	<0.01*
Primary schooling	13 (4.72%)	13 (13.4%)	26 (6.98%)	
Secondary schooling	136 (49.45%)	50 (51.54%)	186 (0.5%)	
Tertiary schooling	124 (45.09%)	32 (32.98%)	156 (41.93%)	
Number of members in family				
1-4	136 (49.45%)	53 (54.63%)	189 (50.8%)	0.658
5-8	126 (45.81%)	31 (31.95%)	157 (42.20%)	
9-12	10 (3.63%)	12 (12.37%)	22 (5.88%)	
13-16	3 (1.09%)	1 (1.03%)	4 (1.07%)	
Significant Family history	192 (69.81%)	66 (68.04%)	258 (69.35%)	0.745
Hypertensives				
Systolic BP >140	90 (32.72%)	20 (20.61%)	110 (29.56%)	<0.05*
Diastolic BP >90	80 (29.09%)	16 (16.49%)	96 (25.80%)	<0.01*
Diabetics	35 (12.72%)	9 (9.27%)	44 (11.82%)	0.367
Addictions				
Smoking status	20 (7.27%)	0	20 (5.37%)	<0.01*
Alcohol consumption	10 (3.63%)	0	10 (2.68%)	0.057
Tobacco consumption	65 (23.63%)	1 (1.03%)	66 (17.74%)	<0.01*
Diet				
Vegetarian	255 (92.72%)	88 (90.72%)	343 (92.20%)	0.528
Mixed	20 (7.27%)	9 (9.27%)	29 (7.79%)	
Physical activity				
Daily	130 (47.27%)	36 (37.11%)	166 (44.62%)	<0.05*
Occasionally	29 (10.54%)	5 (5.15%)	34 (9.13%)	
None	116 (42.18%)	56 (57.73%)	172 (46.23%)	
WHO/ISH risk score				
<10% (Low)	216 (78.54%)	82 (84.53%)	298 (80.10%)	<0.05*
10-20% (Moderate)	22 (8%)	9 (9.27%)	31 (8.33%)	
20-30% (Moderately high)	21 (7.63%)	6 (6.18%)	27 (7.25%)	
30-40% (High)	9 (3.27%)	0	9 (2.42%)	
>40% (Very high)	7 (2.54%)	0	7 (1.88%)	

Age was found to be a significant contributor to the higher score. This risk factor significantly rose between the ages of 70 and 79, with a risk of more than 20% in 35.28% of cases and a very high risk of more than 40% in 23.52% of cases. Only 1.51% of the participants in the age group of 40 to 49 had a risk of >20%. It was found that the prevalence of hypertension increased with age. A majority (64.7%) of participants with systolic blood pressure >140 mmHg were found in the age group of 70 to 79 years (Table 3).

**Table 3:** Correlation between risk factors and age of participants *(p-value<0.05 shows significant difference) WHO: World Health Organization, ISH: International Society of Hypertension, BP: blood pressure

Characteristics	Age (in years)				
	40-49 N=194	50-59 N=97	60-69 N=64	70-79 N=17	P value
WHO/ISH risk score					
<10% (Low)	189 (97.42%)	89 (91.75%)	15 (23.43%)	5 (29.41%)	<0.01*
10-20% (Moderate)	2 (1.01%)	4 (4.12%)	19 (29.68%)	6 (35.29%)	
20-30% (Moderately high)	2 (1.01%)	1 (1.03%)	22 (34.37%)	2 (11.76%)	
30-40% (High)	1 (0.5%)	3 (3.09%)	5 (7.81%)	0	
>40% (Very high)	0	0	3 (4.68%)	4 (23.52%)	
Diabetics	12 (6.18%)	13 (13.4%)	18 (28.12%)	1 (5.88%)	<0.01*
Systolic BP >140 mmHg	48 (24.74%)	22 (22.68%)	29 (45.31%)	11 (64.7%)	<0.01*
Diastolic BP >90 mmHg	64 (32.98%)	19 (19.58%)	9 (14.06%)	4 (23.52%)	<0.01*

A participant's risk of getting CVD was calculated using the WHO/ISH CVD risk prediction charts in a setting where blood cholesterol cannot be measured. The majority of research participants (80.1%) were classified as having a low risk for serious (<10%), followed by 8.33% with a moderate risk (10% to <20%), 7.25% with a moderately high risk (20% to <30%), 2.42% with a high risk (risk 30% to <40%), and 1.88% with a very high risk (>40%). The risk was lower for females compared to males and higher for males. Of the total participants, seven (1.88%) had a CVD risk greater than 40%. Further stratification showed that all of the participants with risk scores >30% had systolic blood pressure >140 mmHg. It was found that there was a significant effect of education, hypertension, smoking, tobacco consumption, and physical activity on CVD (p<0.05). The WHO/ISH risk score was linked with age, sex, the presence or lack of diabetes mellitus and the systolic and diastolic blood pressure ranges and was found to be statistically significant (p<0.05) (Table 4).

**Table 4:** Correlation between WHO/ISH risk score and risk factors *(p-value<0.05 shows significant difference) WHO: World Health Organization, ISH: International Society of Hypertension, BP: blood pressure

	WHO/ISH risk score					
	<10% (Low)	10-20% (Moderate)	20-30% (Moderately high)	30-40% (High)	>40% (Very high)	P value
	N=298	N=31 n(%)	N=27 n(%)	N=9 n(%)	N=7 n(%)	
Systolic BP >140 mmHg	57 (19.12%)	16 (51.61%)	21 (77.77%)	9 (100%)	7 (100%)	<0.01*
Diastolic BP >90 mmHg	75 (25.16%)	4 (12.9%)	7 (25.92%)	5 (55.55%)	5 (71.42%)	<0.01*
Diabetics	23 (7.71%)	5 (16.12%)	10 (37.03%)	5 (55.55%)	1 (14.28%)	<0.01*
Smoking status	13 (4.36%)	3 (9.67%)	3 (11.11%)	1 (11.11%)	0	0.237
Alcohol consumption	7 (2.34%)	2 (6.45%)	1 (3.7%)	0	0	0.930

## Discussion

The data shown above indicate that 80.1% of the population has a CVD risk of less than 10% over the next 10 years. Trideep et al. [15], Madhu et al. [16], Premanandh et al. [9], Ghorpade et al. [17], and Shrivastava et al. [18] arrived at a similar conclusion from their studies independently.

Out of the 74 individuals with a 10-year CVD risk of >10%, 59 (79.72%) were male and 15 (20.27%) were female. In statistical analysis, a high 10-year WHO/ISH CVD risk score was shown to be statistically significant (p<0.05) and related to the male gender. A similar correlation was found in the research by Premanandh et al. who attributed it to higher smoking rates in men [9]. In the current study, all smokers were male. However, smoking and a higher WHO/ISH CVD risk score did not correlate. This could be due to the fact that there was a low proportion of smokers (20 smokers out of 372, that is, 5.37%) in our study. Another reason could be that 85% of those smokers belonged to the age group of 40 to 59 years in which there is no significant difference between WHO/ISH CVD risk prediction graphs of non-smokers and smokers. Except for smoking, all other risk variables used to determine the 10-year CVD risk namely age, diabetes, and systolic blood pressure significantly correlated with the WHO/ISH CVD risk score (p<0.05). Higher diastolic blood pressure and heart rate were also shown to be significantly (p<0.05) linked to higher estimates of 10-year CVD risk. Lack of physical activity was significantly associated (p<0.05) with lower education levels. However, both insufficient physical activity and lower education levels were not related to increased 10-year CVD risk. In a research conducted by Premanandh et al. [9], there was no evident relationship between physical activity and a 10-year CVD risk score.

Distribution of 10-year CVD risk by age shows that a sizable proportion (63.42%) of study participants were aged 40 to 49 years. Most of the participants with a 10-year CVD risk of less than 20% were over 60 years (83.72%). CVD risk was lowest in people from age 40 to 49 years (97.4%). By contrast, those above the age of 60 years had a CVD risk of 20% or less over a decade. The prevalence of hypertension among the study participants was 29.56%, which is close to 22.9% and 36.8%, the prevalence of hypertension in Ahmedabad based on studies done by Parikh et al. [10,19]. It was found that these numbers were comparable to those found in studies by Trideep et al. [15], Mutthunarayanan et al. [20] and Dhungana et al. [21]. The 10-year CVD risk for 33.63% of hypertensives was less than 20%. According to findings by Trideep et al. [15], 48.7% of hypertensives had a risk greater than 20%. The percentage of hypertensives who had a 10-year CVD risk of more than 20% was lower in our study compared to the study by Trideep et al. This could be because 63.63% of the hypertensives in our sample were <60 years old.

Limitations

This was a single-centre open-label all-comer study and hence may be confounded by the selection bias and the results need to be further validated in a larger multicentric study. The majority of the participants in this study were male (73.92%) and less than 50 years of age (52.15%) as most of the time the patient was accompanied by an earning family member. As the first-degree relatives were not aware of their diabetes status we were unable to analyze the same in our study. In developing countries like India, usually, a male family member is the earning member and has more role in the family’s decision making including health-related decisions. Since participants were first-degree relatives of patients, there was significant family history amongst most of the participants. Also, Indians have a large genetic diversity [22]. Hence this study has a limited scope of generalization. Although WHO/ISH risk prediction charts are recommended by WHO as a safe and useful tool for CVD guiding management and treatment decisions for individuals, these charts may not be the most accurate for predicting CVD risk in the Indian population [23-26]. 

## Conclusions

CVD presents a severe threat and is a cause of concern for public health particularly in developing countries. Out of all non-communicable diseases, CVD is the highest contributor even preceding cancer and diabetes. The study aimed to find the prevalence of cardiovascular risk and improve CVD awareness amongst the first-degree relatives of patients who were ≥40 years of age using WHO/ISH risk prediction charts. Majority of the study participants were in the low-risk category (<10%). Even though the prevalence of hypertension was 29.56%, only 11.55% of subjects had a 10-year risk of 20% or higher. High risk was related to older age, male sex, diabetes status, and higher systolic and diastolic blood pressure levels. Since age and sex are non-modifiable risk factors for heart disease, interventions such as managing blood pressure, smoking cessation and diabetes management can reduce the 10-year and lifetime risk of heart-related disease. WHO/ISH risk prediction charts are a useful tool for rapid assessment and classification of the population, which in turn helps to design specific therapies for the patients.
